# A Review on Increasing the Targeting of PAMAM as Carriers in Glioma Therapy

**DOI:** 10.3390/biomedicines10102455

**Published:** 2022-10-01

**Authors:** Xingyue Li, Wenjing Ta, Ruochen Hua, Jihong Song, Wen Lu

**Affiliations:** School of Pharmacy, Health Science Center, Xi’an Jiaotong University, No. 76, Yanta West Road, Xi’an 710061, China

**Keywords:** PAMAM dendrimers, nanodrug delivery systems, glioma, brain targeting, blood–brain barrier

## Abstract

Glioma is an invasive brain cancer, and it is difficult to achieve desired therapeutic effects due to the high postoperative recurrence rate and limited efficacy of drug therapy hindered by the biological barrier of brain tissue. Nanodrug delivery systems are of great interest, and many efforts have been made to utilize them for glioma treatment. Polyamidoamine (PAMAM), a starburst dendrimer, provides malleable molecular size, functionalized molecular structure and penetrable brain barrier characteristics. Therefore, PAMAM-based nanodrug delivery systems (PAMAM DDS) are preferred for glioma treatment research. In this review, experimental studies on PAMAM DDS for glioma therapy were focused on and summarized. Emphasis was given to three major topics: methods of drug loading, linkers between drug/ligand and PAMAM and ligands of modified PAMAM. A strategy for well-designed PAMAM DDS for glioma treatment was proposed. Purposefully understanding the physicochemical and structural characteristics of drugs is necessary for selecting drug loading methods and achieving high drug loading capacity. Additionally, functional ligands contribute to achieving the brain targeting, brain penetration and low toxicity of PAMAM DDS. Furthermore, a brilliant linker facilitates multidrug combination and multifunctional PAMAM DDS. PAMAM DDS show excellent promise as drug vehicles and will be further studied for product development and safety evaluation.

## 1. Introduction

**Figure 1:**
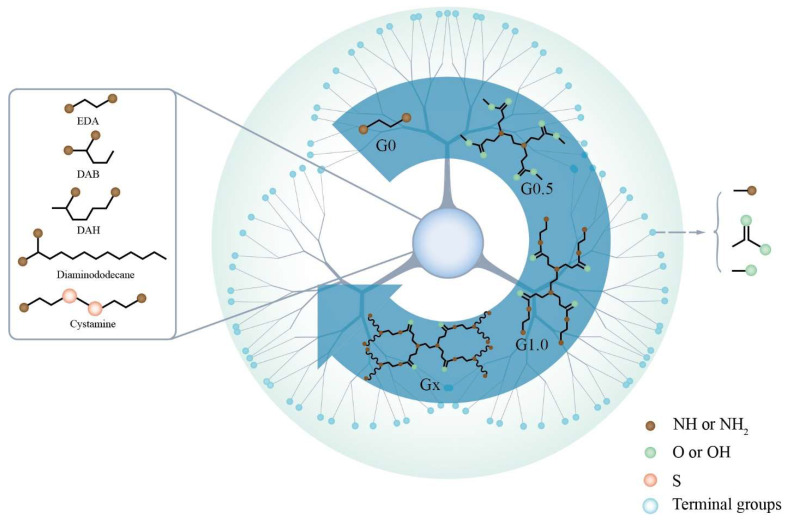
Schematic diagram of the structure of PAMAM. Schematic showing the chemical structure of PAMAM, including the core, terminal groups and different generations. EDA: ethylenediamine; DAB: diaminobutane; DAH: 1,5-diaminohexane.

In 1985, the America scientist, Tomalia, synthesized polyamidoamine dendrimers (PAMAM) for the first time by Michael’s addition reaction using ammonia or ethylenediamine as the starting monomer and adding methyl acrylate [1]. Since then, PAMAM have attracted increasing attention, and their structures, derivatives, modifications and applications have been reported more and more frequently. As basic building blocks, PAMAM consist of an initiator core, multiple branches and terminal groups (Figure 1).

Diamine is essential for the core of PAMAM; some classic cores, such as ethylenediamine (EDA), diaminobutane (DAB), 1,5-diaminohexane (DAH) and cystamine all possess a diamine structure. The difference among cores is the alkyl chain, which affects the stability of PAMAM. If cystamine is the core, a disulfide bond will break in the presence of a reducing agent (e.g., glutathione or β-mercaptoethanol) [2]. Branches consisting of multiple repeat units are vital. This is because many branches wind and form a hydrophobic region called a cavity [3], which is involved in encapsulating a hydrophobic drug. The number, size and hydrophobicity of the cavity are affected by the branches [3,4]. High-generation PAMAM possess more than one cavity owing to their multiple branches, whereas low-generation PAMAM may not even have a cavity. Branches that possess a long alkyl chain lead to a larger cavity due to the decreasing fold. Terminal groups on the PAMAM may be amino groups, carboxyl groups or hydroxyl groups. The type and number of terminal groups affect the linking of drugs or targeting ligand and the toxicity of the PAMAM. Taking amino group terminal groups as an example, PAMAM-NH_2_ has stronger cytotoxicity than PAMAM-COOH and PAMAM-OH, because the terminal possesses excessive positive charge and easily reacts with cell membranes, causing cytotoxicity [5].

PAMAM have nanoscale size, reactive terminal groups and a hydrophobic cavity, thus, are used in many fields, including in the environmental field and in medicine [6]. From an environmental perspective, PAMAM take advantage of the active terminal groups to treat water and rapture carbon dioxide [7]. From a medical perspective, PAMAM are principally used to deliver genes or drugs to increase solubility or target ability or promote absorption for diagnosing and treating disease. In 1995, for the first time, researchers developed the concept of using PAMAM as DNA carriers [8]. Since then, growing attention has been paid to the application of PAMAM as carriers in medicine to treat various diseases, such as prostate cancer, breast cancer and glioma [9,10,11].

In this review, we mainly focus on the application of PAMAM in the treatment of glioma. A glioma is an aggressive, malignant tumor that damages human health. After surgery, drug treatment is necessary for glioma patients to decrease the possibility of recurrence. Challenges can occur during the process of drug delivery, such as when crossing the blood–brain barrier (BBB) and blood–brain–tumor barrier (BBTB), and diffusion of drugs in the brain [12]. The existing intercellular tight junctions (TJ) and efflux transporters on the BBB restrict the entry of most drugs into the brain [13]. Using PAMAM, which modify brain-targeting ligands, as carriers helps drugs to easily cross the BBB and overcome poor brain distribution. A BBB penetration experiment demonstrated that the BBB transport ratio of doxorubicin (DOX) delivered by ANG-PEG-PAMAM was approximately 17% in human brain microvascular endothelial cells (HBMVECs), which was dramatically higher than that of free DOX [14]. In C6 glioma cells, the cellular uptake behavior was observed, and the uptake amount of G5 PAMAM-PEG-Angiopep-2/TRAIL NPs and G5 PAMAM-PEG-Tf/TRAIL NPs was higher than that of naked TRAIL or brain-targeting, ligand-modified PAMAM-carried TRAIL. Additionally, previous studies determined that G5 PAMAM-PEG-Angiopep-2/TRAIL NPs and G5 PAMAM-PEG-Tf/TRAIL NPs exhibited relatively stronger accumulation in the brain tissue for brain-tumor-bearing mice and C6-glioma-bearing rats compared with in other organs [15,16]. The BBTB formed in the process of tumor deterioration limited the drugs that enter the tumor [12]. Using PAMAM as carriers and modifying them with ligands to target overexpressing substances in the tumor help the drugs to cross the BBTB. Using flow cytometry, it was observed that the uptake amount of EGFR ASODN delivered by G5 PAMAM-FA was two times that delivered by oligofectamine in C6 glioma cells [17]. A U251 glioma xenograft model showed a 70-fold difference in the tumor growth rate between the treatment group with psiRNA-EGFR delivered by Tat-BMPs-PAMAM and the PBS treatment group [18]. In these studies, modified PAMAM can be considered promising carriers in delivering drugs for glioma treatment.

## 2. Brain-Targeted Drug Delivery System Based on PAMAM

The drug loading method, the ligand-modified PAMAM and the linker between the PAMAM and drugs or ligands are considered to be important for constructing an effective and safe brain-targeted drug delivery system (DDS) based on PAMAM.

## 3. Methods of Drug Loading

**Figure 2:**
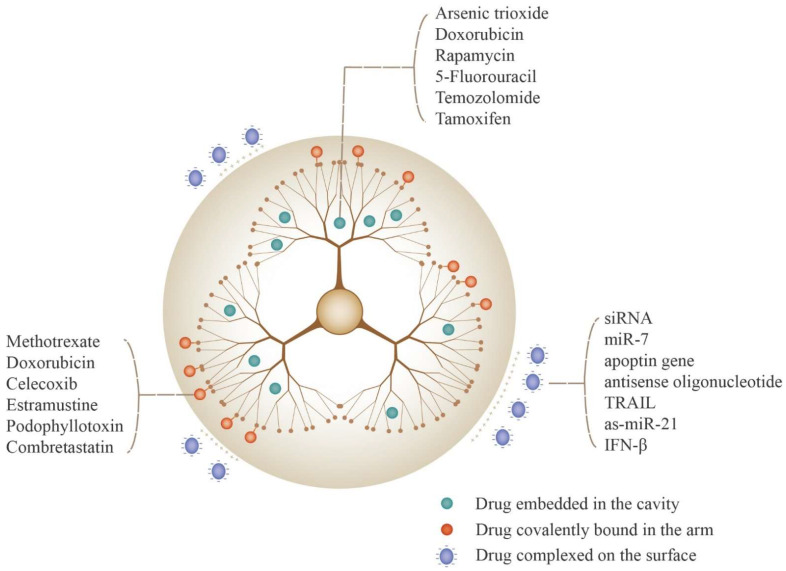
Methods of loading drugs in PAMAM DDS. Drugs can be embedded in the PAMAM’s cavity or complexed on the surface of PAMAM or covalently bound in the arm of PAMAM. SiRNA: small interfering RNA; miRNA-7: microRNA-7; TRAIL: tumor-necrosis-factor-related apoptosis-inducing ligand; IFN-β: interferon-β.

Many studies have shown that there are three main methods of drug loading based on the structure and properties of the drugs, which are embedded in the cavity of PAMAM, complexed on the surface of PAMAM and covalently bound in the arm of PAMAM, and the PAMAM (Figure 2). The differences of these methods are shown in Table 1.

### 3.1. Embedded in the Cavity of PAMAM

Some hydrophobic molecules, such as arsenic trioxide (ATO), DOX and rapamycin, can be encapsulated in the inner cavity of PAMAM by hydrophobic interaction to improve release behavior and brain targeting. Compared with ATO sol, ATO embedded in PEG-PAMAM showed a slow-release characteristic without sudden release [19]. It was demonstrated that the tumor accumulation of embedded ATO was six-fold greater than that of free ATO [20]. DOX processes cardiotoxicity; its clinical application as an antitumor drug is limited. In addition, the molecular weight of DOX is large, further preventing it from crossing the BBB. Hence, a targeting carrier is needed to load DOX and increase its ability to cross the BBB and target tumors. He et al. [21] embedded DOX hydrochloride by the equilibrium dialysis method in a dual-targeting carrier, which was PAMAM-PEG-WGA-Tf. It was proved that embedded DOX enhanced BBB transport and glioma accumulation through WGA-induced adsorptive endocytosis and Tf-induced, receptor-mediated transport action. Similar conclusions were obtained for embedded DOX hydrochloride in different modified PAMAM [14,22]. Therefore, it is wise to embed drugs in the PAMAM’s cavities when using drugs with antitumor activity but difficulty in penetrating the BBB and low water solubility.

### 3.2. Complexed on the Surface of PAMAM

Some RNA, DNA or oligonucleotides, such as small interfering RNA (siRNA), miR-7 and apoptin, demonstrate glioma therapy potential by conjugating the surface of PAMAM [23,24,25]. Owing to the surface of PAMAM having positively charged terminal amino groups and drugs with a phosphate group that are negatively charged, the preparation of drug–PAMAM complexes based on an appropriate N/P ratio can be implemented via electrostatic interaction. A PAMAM-FHR/pJDK-apoptin (F, H and R refer to phenylalanine, histidine and arginine, respectively) complex was prepared with a weight ratio of apoptin and PAMAM of 2. Conjugating apoptin on the PAMAM-FHR allowed it to more easily escape from the endosome and be released into the cytosol to induce cell apoptosis [26]. Other drugs and their optimum ratios for increasing targeting are shown in Table 2.

### 3.3. Covalently Bound in the Arm of PAMAM

Drugs, such as methotrexate (MTX), DOX and celecoxib, can be linked in the branches of PAMAM to increase brain and tumor targeting. Linking drugs in the arm of PAMAM fully utilizes the reactive behavior between amino groups of PAMAM and the active groups of drugs. To ensure that the chemo-action between drug and amino groups of PAMAM can occur, some reagents to activate the drugs or PAMAM are typically used. Succinic anhydride is a feasible reagent and has been used to activate DOX [36], celecoxib [37] and others. There are some reagents that are selectable in activating drugs, DOX for instance; cis-aconitic anhydride, BMPH (*N*-(β-maleimidopropionic acid) hydrazide HCl) and 2-iminothiolane are also practicable, activated reagents [38,39,40]. In addition to activating drugs, activating PAMAM is also vital for linking. SPDP, N-succinimidyl-3-(2-pyridyldithio) proprionate, a cross-linking agent, can activate PAMAM. In a study by Gong Wu et al. [41], G5 PAMAM were activated by SPDP and covalently bound with MTX that had reacted with 1-(3-dimethylaminopropyl)-3-ethylcarbodiimide (EDC) and N-hydroxysuccinimide (NHS). Cetuximab (IMC-C225) was subsequently used to modify PAMAM to increase the specific targeting of tumor cells. In another study [42], thiolated DOX was introduced to the exterior of PAMAM via reaction with SPDP-activated PAMAM and then modified with a target ligand (angiopep-2). It was found that DOX linked in the arm of PAMAM exhibited higher BBB transport ratios (over 11.9%). It is important to select non-toxic and stable activating reagents according to the active group in the drug, the terminal group of the PAMAM and the characteristics of the glioma microenvironment. This choice also needs to ensure ease of reaction and stability and site-specific release of newly generated bonds.

### 3.4. Linkers between Drug/Ligands and PAMAM

**Figure 3:**
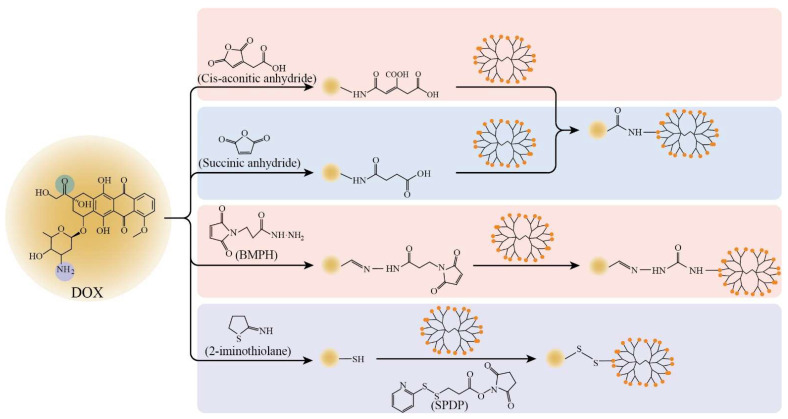
Schematic diagram of the formation process of different linkers between drugs and PAMAM. Different background colors represent the synthesis of linkers with different release properties: pink for acid-sensitive, blue for non-acid-sensitive and purple for GSH-sensitive. DOX is taken as an example of a drug. DOX: doxorubicin; BMPH: N-(β-maleimidopropionic acid) hydrazide; SPDP: N-succinimidyl-3-(2-pyridyldithio) proprionate.

The linker determines the strength of drug binding with modified PAMAM and the release of drug and further affects the targeted delivery of a drug. First, to accomplish the linkage between drug/ligands and PAMAM, a linker with at least two active groups at the end of the chain is required. Second, to satisfy the targeting performance, it is critical that the linker breaks in response to specific conditions. The formation process of different linkers using DOX as an example is shown in Figure 3.

Cis-aconitic anhydride has a five-membered, unsaturated, oxygen-containing ring, which opens in a phosphate buffer containing EDC, releasing two active carboxyl groups. One carboxyl group can react with the amino group of the drugs, and another can react with the amino group of the PAMAM. In the presence of EDC, PEG-PAMAM-cis-aconityl-DOX conjugate (PPCD) was obtained by adding cis-aconitic anhydride [30]. It was found that the content of DOX released from PPCD at pH 4.5 was 120 times higher than that at pH 7.4 in 96 h, indicating that the cis-aconityl linker possesses an acid-sensitive trait. An acid-sensitive trait is propitious for enhancing tumor targeting because lysosome is an acidic environment, whereas plasma is basic. Under low pH in lysozyme, the carriers with an acid-sensitive linker are prone to lysis and release large quantities of drugs [43].

Succinic anhydride has the structure of a five-membered, oxygen-containing ring, which is opened in the presence of triethylamine; two active carboxyl groups are released, which can react with the amino or hydroxyl group at the end of the drug or PAMAM. PEG-PAMAM-succinic-DOX conjugate (PPSD) was obtained by adding succinic anhydride, which first reacted with the amino group on DOX, and then reacted with the amino group of PEG-PAMAM in the presence of EDC and NHS [38]. However, PPSD is not acid sensitive; the amount of DOX released from PPSD was lower than that from PPCD, resulting in weak antitumor activity [43,44]. In the study of Ugil Hussainsk et al., D-EM and D-PODO were prepared by adding succinic anhydride, which first reacted with the hydroxyl group of estramustine (EM) or podophyllotoxin (PODO) and then reacted with the hydroxyl group of the PAMAM. It was found that D-EM and D-PODO further decreased the viability of glioma cells compared with free drugs [45].

Compounds with an acyl hydrazone group and a five-membered ring of an unsaturated cyclic diketone containing nitrogen have also been used to link DOX, and modified G4 PAMAM take advantage of its structure of imine and active carbonyl. One feasible approach is created by the presence of anhydrous methanol and trifluoroacetic acid, where BMPH (heterobifunctional hydrazide linkers) can convert carbonyl on the side chain of DOX into imine and introduce another active carbonyl of BMPH [39]. Li et al. [46] introduced DOX into PAMAM via the method above and determined that more DOX was released at pH 4.5 (32%) than at pH 7.4 (6%) in 24 h. They demonstrated that, similarly to cis-aconityl acid linkers, linking via acyl hydrazone bonds presents acid-sensitive break behavior and increases the glioma-targeting potential of DOX.

GSH (glutathione)-sensitive linkers are also recognized for improving the glioma targeting of drugs that covalently bind with PAMAM, because the intracellular concentration of GSH is much higher in the glioma site than the extracellular concentration [47]. A disulfide bond is a GSH-responsive linker, breaking with the increasing concentration of GSH. Xu et al. [42] used 2-iminothiolane (a thiolated reagent) to react with DOX and introduced a sulfhydryl at the terminal amino group of DOX, then DOX-SH connected with SPDP-activated PAMAM and built a disulfide bond to achieve the link of DOX and the PAMAM. In the group with a GSH concentration of 10 mM, the cumulative release of DOX was over ten times that of the control group without GSH, which proved that DOX from this carrier presents a GSH-responsive behavior and lower cytotoxicity compared with free DOX in brain microvascular endothelial cells (BMVECs).

### 3.5. Ligands of Modified PAMAM for Brain Targeting

**Figure 4:**
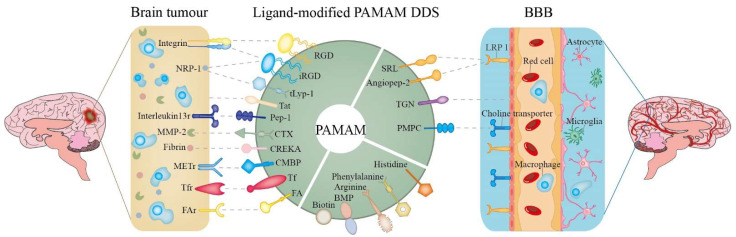
Schematic representation of targeting ligands and their receptors or targeting sites on the PAMAM DDS. NRP-1: neuropilin 1; MMP-2: matrix metalloproteinase-2; METr: mesenchymal transition factor receptor; Tfr: transferrin receptor; FAr: folic acid receptor; CTX: chlorotoxin; Tf: transferrin; FA: folic acid; PMPC: poly (2-methacryloyloxyethyl phosphorylcholine); BMP: bacterial magnetic nanoparticles; LRP 1: low-density lipoprotein receptor-related protein 1.

To further increase the glioma targeting of PAMAM delivery systems, some ligands, such as transferrin (Tf) and angiopep-2, have been used to modify PAMAM. Here, the ligands of modified PAMAM in the literature from 2006 to 2021 are summarized in Figure 4. These include amino acids, peptides, proteins, vitamins and others.

### 3.6. Amino Acids

Amino acids are widely used to modify PAMAM for research on glioma therapy, as introducing them can improve PAMAM’s biocompatibility. In the process of studying glioma therapy, the first amino acid used to modify PAMAM was arginine. Arginine is a basic amino acid which can neutralize the positive charges on the surface of PAMAM and decrease the density of positive charges to reduce toxicity as well as increase targeting. G4 PAMAM-R as a gene carrier significantly improved the expression level of target protein (IFN-β) and possessed the specifically ability to kill in Neuro2A (mouse neuroblastoma N2a cells) and U87 MG cells (U87 malignant glioma cells) [33]. In addition to arginine, previous research investigated PAMAM modified by lysine and histidine as ligands. G4 PAMAM-H-R as a gene carrier has lower cytotoxicity, higher cellular uptake and a more excellent transfection efficiency than PAMAM-H-K (K refers to lysine) and unmodified PAMAM in GBL-14 cells [48]. Bae et al. [25], based on the Alexa Fluor 488-labeled PAMAM-H-R/apoptin observed in the cytoplasm, speculated that histidine-modified G4 PAMAM-R results in enhanced “proton sponge effects”, leading to more drugs (apoptin) being able to escape from the endosome to the cytoplasm and further enter the nucleus of glioma cells to induce apoptosis. Phenylalanine’s structure contains a phenyl group, which enhances the lipid solubility of PAMAM-H-R and the binding of PAMAM-FHR to the lipid bilayer, promoting cell internalization. Additionally, through increasing the proton buffer ability, the expression of apoptin delivered by G4 PAMAM-F-H-R was enhanced in the GBL-14 cell line [26].

### 3.7. Peptides

Since 2009, peptides have been used to modify PAMAM to increase targeting to glioma as most of them can bind with receptors with specific distributions to enhance targeting.

RGD (arginine-glycine-aspartic acid) can bind to integrin proteins with high affinity which overexpress on the surface of tumor cells (especially cancer-related integrin, e.g., αvβ3) [49]. RGD-modified G5 PAMAM showed increasing delivery ability compared with naked PAMAM in U87 MG cells [23,50]. In the studies of Waite et al. [23,50], G5 PAMAM-RGD conjugates overcame the issue of low permeability of PAMAM and improved the capacity of delivering drugs to gliomas by interfering with the binding affinity of integrin–ECM. Zhang et al. [43] also drew similar conclusions using in situ mouse models of C6 glioma. In another study by Wang et al. [44], RGD was replaced with internalized RGD (iRGD) to modify the PAMAM. iRGD peptides can bind to integrin as RGD and can also combine with NRP-1 (neuropilin-1) to enhance the permeability of tumors. They demonstrated that the iRGD-mediated delivery system possessed stronger penetration and higher accumulation in brain tumors compared with traditional, RGD-mediated delivery systems [44]. TGN (consisting of 12-amino-acid TGNYKALHPHNG) was obtained through phage display technology and was proved to be a highly effective brain-targeting peptide [51]. In 2020, Shi et al. [20] developed a dual-targeting delivery system (iRGD/TGN-PEG-G5 PAMAM) by introducing iRGD and TGN simultaneously.

Tat peptide (CGRKKRRQRRRK), being a trans-activating transcriptional activator protein and Tat-mediated delivery system that effectively passes through biological membranes, was used to further promote intracellular delivery of drugs; Han et al. developed a novel delivery system, Tat-BMPs-PAMAM, which exhibited great antitumor ability and transfect efficiency as a gene carrier [18,52]. Pep-1 (CGEMGWVRC) can target IL-13Rα2 (interleukin-13 receptor alpha 2), which is overexpressed in glioma cell lines, and can overcome the interference of the BBTB by promoting IL-13Rα2-mediated endocytosis [53]. It was found by the real-time imaging of intracranial U87MG tumor-bearing mice that the accumulation of Pep-PEG-G5 PAMAM at glioma sites is significantly increased [54]. tLyp-1 (CGNKRTR) is a type of tumor-homing peptide which can bind to neuropilin-1 (NRP-1) to facilitate the penetration of drugs in tumors [55]. Jin et al. [56] developed a novel drug delivery system by modifying PAMAM with tLyp-1 and confirmed that this drug delivery system could effectively cross the BBB and inhibit glioblastoma.

Except for tumor-penetrating peptides, there are some peptides that can be introduced into PAMAM to increase tumor targeting, such as chlorotoxin (CTX) and CREKA (cysteine-arginine-glutamine-lysine-alanine). CTX can bind to matrix metalloproteinase-2 (MMP-2) endopeptidase with high affinity, and this enzyme is preferentially upregulated in glioma. G5-PAMAM-PEG-CTX as a delivery system can remarkably increase cellular uptake of drugs in C6 glioma cells [30]. CREKA, a fibrin-binding peptide, can target abundant fibrin in GBM and enhance retention in tumors. In a study by Zhao et al. [57], CREKA was conjugated with G5 PAMAM to develop a new peptide-mediated drug delivery system.

Angiopep-2 is another peptide extensively used in modifying PAMAM to increase brain targeting [58,59]. Angiopep-2 can target low-density lipoprotein receptor-related protein 1 (LRP1), which is expressed in brain capillary endothelial cells and neuroglial cells [60]. G5 PAMAM-PEG-angiopep/DNA NPs have demonstrated higher distributions at brain tumor sites compared with G5 PAMAM-PEG/DNA NPs and G5 PAMAM/DNA NPs [15]. Studies have also shown that the binding of angiopep-2 to LRP1 further promotes LRP-mediated endocytosis, enhances the accumulation of drugs in tumor and facilitates BBB penetration [14,42]. Yan et al. [61] developed a targeting nanoprobe that simultaneously labeled RGDyk and angiopep-2 on G5 PAMAM and confirmed this as a non-invasive visualization technique for brain tumors via targeting αvβ3 integrin and LRP1. In addition to angiopep-2, SRL (serine-arginine-leucine) peptides can also target LRP receptors. A novel nano-carrier (PAMAM-PEG-SRL) was developed and exhibited excellent transfect efficacy [62].

cMBP is a polypeptide-targeting mesenchymal transition factor (MET) receptor, which can compete with hepatocyte growth factor (HGF) by binding to MET sites to further block the RAF-MEK-ERK (MAPK) and PI3K-Akt signal pathways. Additionally, METs are abnormally activated in most glioma patients; hence, it is necessary to introduce cMBP to modify PAMAM. CBMP-PEG-G4 PAMAM have been proven to effectively decrease proliferation and invasion of U87MG cells [63]. KRRR peptide is a nuclear localization signal. Bae et al. [35] modified G3 PAMAM with KRRR peptide and found that this can increase nuclear localization by facilitating nuclear pore complex (NPC)-mediated active transport.

### 3.8. Proteins

Tf, specifically binding with Tf receptors that overexpress on BMVECs and tumor cells via Tf receptor-mediated endocytosis, can be used to cross the BBB and target tumors. In 2011, reports indicated that Tf-modified PAMMAM increase targeting in glioma therapy. They showed that G4 PAMAM-PEG modified with Tf had stronger transport capacity across the BBB compared with that which was unmodified by Tf when delivering the drugs [16,46]. Furthermore, the viability of the C6 glioma cells was 14.5% for G4 PAMAM-PEG-WGA-Tf-DOX, 21.3% for G4 PAMAM-PEG-Tf-DOX and 23.7% for G4 PAMAM-PEG-WGA-DOX, which indicates that G4 PAMAM-PEG-WGA-Tf had stronger cytotoxicity [21]. Similar to Tf, lactoferrin, insulin and LRP all possess specific receptors on BMVECs. However, low expression level of receptors on BMVECs or the instability of ligand protein may limit their direct application in glioma research.

### 3.9. Vitamins

Vitamins, especially water-soluble vitamins, including folic acid (FA) and biotin, are used as ligands to modify PAMAM to enhance targeting in glioma therapy. FA (i.e., vitamin B9) has been broadly applied to modify PAMAM in glioma research since 2010. FA specifically binds to FA receptors, which are overexpressed on malignant tumor cells, and then enters the tumor cells through FA-receptor-mediated endocytosis. A FA-G5 PAMAM–drugs complex (drugs refers to antisense oligonucleotides, DOX) greatly inhibited the growth of C6 glioma cells [17,22]. This finding correlated with a study by Liu et al. [24], who used U251 human glioma cells. Biotin (i.e., vitamin B7) is another vitamin that has been used to modify PAMAM to target glioma [64,65]. Biotin can promote the growth of cells and rapidly proliferate tumor cells, requiring additional biotin. Uram et al. [66,67] demonstrated that using biotinylated G3 PAMAM as carriers resulted in higher cytotoxicity to the human glioblastoma cell line (U-118 MG) than that achieved by free drugs.

### 3.10. Others

Acetylcholine and *N*-acetylcholine receptors are highly expressed in brain tissue. Poly (2-methacryloyloxyethyl phosphorylcholine) (PMPC) has a similar structure to the specific ligands of these receptors, thus, may specifically recognize or bind to the receptors. Therefore, PMPC-modified PAMAM DDS can increase the brain transport of drugs through the mediation of PMPC. In addition to the structure of PMPC being similar to cellular phospholipid layers, the addition of PMPC on PAMAM can also reduce cytotoxicity. Ban et al. [68] proved that PAMAM modified with bifunctional ligand PMPC were a novel and effective brain-targeted delivery system.

Bacterial magnetic nanoparticles (BMP) are a magnetic substance covered with a lipid membrane and are used to modify PAMAM. Based on the controlled size and particular biocompatibility and magnetic targeting of BMPs, Han et al. [18] built an efficient delivery system, Tat-BMPs-G3 PAMAM. They found that this system significantly enhanced transfect efficiency in U251 human glioma cells [18,52].

Some sugars, such as chitosan, glucose or mannose, can also be considered to increase glioma-targeting ligands [69,70,71]. Sharma et al. [71] demonstrated chitosan-modified PAMAM delivering temozolomide in the brain achieve a higher distribution than pure drugs by opening the TJ between cells. It was proved that the accumulation of glycosylated PAMAM-OH was over 8-fold that of the non-modified tumor, and it enhanced tumor-associated macrophages (TAMs) targeting [69]. 

## 4. Strategies of PAMAM Drug Delivery Systems for Glioma Therapy

Up to now, the treatment of glioma has been a research hotspot. Although treatment means and drugs for glioma continue to spring up, therapies with maximum efficacy and minimal side effects/toxicity are desired and have been explored. PAMAM provide malleable molecular size, functionalized molecular structure and penetrable BBB molecular characteristics, and PAMAM DDS are a preferred choice for glioma therapy research. As a proof of concept, well-designed PAMAM DDS are constructed from three aspects.

Firstly, an appropriate method of drug loading is a prerequisite. The physicochemical properties of drugs can provide some insights. Hydrophobic, small-molecule drugs are more easily encapsulated in PAMAM’s cavities through physical adsorption or intermolecular interaction. Electrically charged amphoteric drugs can form electrostatic binding with PAMAM by adjusting the pH of the coexisting solution. In addition, the reactive groups of the drug molecule structure provide a means of covalently binding to PAMAM via an appropriate linker. Therefore, it is important to purposefully understand the physicochemical properties and structural characteristics of the drug for the selection of appropriate drug loading methods in PAMAM DDS.

Secondly, ligands are the key to the functionalization of PAMAM DDS. Many active terminal groups in the PAMAM’s molecules facilitate the realization of functionalization. Brain targeting of PAMAM DDS can be achieved by matching specific ligands with characteristic targets of glioma or brain tissue. For example, it has been clearly reported that RGD and CTX can specifically recognize overexpressed integrin and MMP-2 in glioma, as well as angiopep-2, which can promote the gathering of PAMAM DDS in the brain due to the high expression of LRP1 in the brain microvascular system. Some ligands, such as TGN peptide and β-cyclodextrin (β-CD), have also been experimentally found to be capable of brain targeting; however, their mechanisms and targets still need to be clarified [28,51]. Of course, more targets will be reported as the biology of glioma progression and the microstructure of BBB are elucidated. Aiming to improve the transporters or intercellular tight proteins of BBB, ligands were explored to modify PAMAM DDS. This approach was designed to achieve brain targeting by further enhancing the brain penetration of PAMAM DDS. Tf increases brain penetration levels of PAMAM DDS by increasing Tf-receptor-mediated endocytosis. There are also ligands that are more likely to reduce the systemic toxicity of PAMAM DDS. PAMAM are known to have a high surface charge density and may produce biostatic effects in vivo. Therefore, ligand modifications, such as modification of arginine, reduce the charge density of PAMAM and increase compatibility, which is conducive to well-designed PAMAM DDS. In recent years, smart PAMAM DDS have attracted great interest from researchers. PAMAM DDS modified by iRGD and TGN have the dual functions of brain targeting and promoting brain penetration, and DDS modified by FA and borneol can target the brain while reducing the toxicity of DDS [20,72]. In addition, G4 PAMAM-PEG-Tf-WGA realizes the multiple functions of targeting, promoting penetration and reducing toxicity [59]. With the continuous development of new ligands and the diversification of ligand combinations, some novel PAMAM DDS will be designed and constructed.

Finally, a linker is indispensable for the binding of drugs or ligands to PAMAM, especially covalent binding. A brilliant linker should be able to connect drugs to the PAMAM. Moreover, it can avoid the steric hindrance of PAMAM and realize multiple connections. This facilitates multidrug combinations and multiple types of functional PAMAM DDS. Furthermore, based on the characteristics of the microenvironment of the lesion area, the linker of the responsive fracture is more desired. It can help PAMAM DDS achieve more types of function, such as pH sensitivity, enzyme responsiveness and GSH responsiveness.

## 5. Conclusions

PAMAM DDS have been explored for a wide range of biomedical applications and for the delivery of many drugs. In this review, PAMAM were considered as delivery vehicles for glioma drugs. Many reported experimental studies have provided beneficial implications for brain-targeted PAMAM DDS to improve effectiveness, decrease side effects and enhance brain permeability. Although further clinical use of PAMAM DDS may be hindered by the undefined cytotoxicity of PAMAM, the exploration of PAMAM DDS for drug delivery selectivity and precision diagnostic applications will continue as the biodegradability, attenuated toxicity and multifunctionality of PAMAM develop.

## Figures and Tables

**Table 1:** Advantages and disadvantages of drug loading methods.

Methods	Advantages	Disadvantages
Embedded in the cavity of PAMAM	Suitable for small-molecule, hydrophobic drugs, such as DOX, ATO; Improves solubility of hydrophobic drugs; Avoids burst release; Enhances bioavailability of insoluble drugs.	Not suitable for small-molecule hydrophilic or macromolecular hydrophobic drugs; Low drug responsiveness to specific environments.
Complexed on the surface of PAMAM	Avoids nucleic acid (such as RNA) degradation by nucleases; Decreases the surface charge of PAMAM; Improves transfection efficiency; Enhances brain targeting.	Only suits ionizable drugs, such as ibuprofen, siRNA; Instability of drug–PAMAM complexes once environment, such as pH, changes.
Covalently bound in the arm of PAMAM	Suitable for drugs with reactive groups; Increases the stability of binding drugs; Responsive and controlled drug release.	Decreases water solubility of PAMAM when bound with hydrophobic drugs; Requires activating reagents and specific reaction conditions.

**Table 2:** Optimum ratios of carrier: drugs.

Drugs	Carriers	Optimum Ratios (Carrier: Drugs)	Date	Reference
ASODNs	G5 PAMAM	1:16	2009	[17]
anti-GFP siRNA	G5 PAMAM-RGD	15:1	2009	[23]
anti-GFP siRNA	G5 PAMAM-AC(20,40,60)	10:1	2009	[27]
psiRNA-EGFR	G3 PAMAM -Tat-BMPs	12.5:1	2010	[18]
VEGF siRNA Bcl-2 siRNA	Au-G5 PAMAM-β-CD	1:5	2018	[28]
SiRNA	(G2-G4) PAMAM-Bis-MPA	2.5:1	2018	[29]
TRAIL	G5 PAMAM-PEG-CTX	3:1	2010	[30]
TRAIL	G5 PAMAM-PEG-Angiopep	3:1	2011	[15]
pORF-hTRAIL	G5 PAMAM-PEG	3:1	2016	[16]
as-miR-21	G5 PAMAM-5-FU	16:1	2012	[31]
miR-21i	G5 PAMAM	16:1	2012	[32]
pORF-IFN-β plasmid	G4 PAMAM-R	4:1	2013	[33]
miR-7	PAMAM-FA	16:1	2013	[24]
pJDK-apoptin	G4 PAMAM, G4 PAMAM-R, G4 PAMAM-H-R, G4 PAMAM-H-K	2:1	2017	[34]
	G4 PAMAM-FHR	2:1	2019	[26]
	G3 PAMAM-KRRR	4:1	2021	[35]

Note: The optimum ratios of the complexes are charge ratios (N/P) that were calculated as a ratio of the number of primary amines in the polymer carrier to the number of anionic phosphate groups in the RNA or DNA.

## Data Availability

Not applicable.
